# Prognostic factors associated with mortality of drug-resistant *Acinetobacter baumannii* ventilator-associated pneumonia

**DOI:** 10.1186/s40560-015-0077-4

**Published:** 2015-03-02

**Authors:** Juthamas Inchai, Chaicharn Pothirat, Chaiwat Bumroongkit, Atikun Limsukon, Weerayut Khositsakulchai, Chalerm Liwsrisakun

**Affiliations:** Division of Pulmonary, Critical Care and Allergy, Department of Medicine, Faculty of Medicine, Chiang Mai University, Chiang Mai, 50200 Thailand; Department of Medicine, Nakornping Hospital, Chiang Mai, Thailand

**Keywords:** Ventilator-associated pneumonia, Extensively drug-resistant *A. baumannii*, Pandrug-resistant *A. baumannii*, Prognostic factor, Mortality

## Abstract

**Background:**

Ventilator-associated pneumonia (VAP) caused by drug-resistant *Acinetobacter baumannii* is associated with high mortality in critically ill patients. We identified the prognostic factors of 30-day mortality in patients with VAP caused by drug-resistant *A. baumannii* and compared survival outcomes among multidrug-resistant (MDR), extensively drug-resistant (XDR) and pandrug-resistant (PDR) *A. baumannii* VAP.

**Methods:**

A retrospective cohort study was conducted in the Medical Intensive Care Unit at Chiang Mai University Hospital, Thailand. All adult patients diagnosed with *A. baumannii* VAP between 2005 and 2011 were eligible. Univariable and multivariable Cox’s proportional hazards regression were performed to identify the prognostic factors of 30-day mortality.

**Results:**

A total of 337 patients with microbiologically confirmed *A. baumannii* VAP were included. The proportion of drug-sensitive (DS), MDR, XDR, and PDR *A. baumannii* were 9.8%, 21.4%, 65.3%, and 3.6%, respectively. The 30-day mortality rates were 21.2%, 31.9%, 56.8%, and 66.7%, respectively. The independent prognostic factors were SOFA score >5 (hazard ratio (HR) = 3.33, 95% confidence interval (CI) 1.94–5.72, *P* < 0.001), presence of septic shock (HR = 2.66, 95% CI 1.71–4.12, *P* < 0.001), Simplified Acute Physiology Score (SAPS) II >45 (HR = 1.58, 95% CI 1.01–2.46, *P* = 0.045), and inappropriate initial antibiotic treatment (HR = 1.53, 95% CI 1.08–2.20, *P* = 0.016).

**Conclusions:**

Drug-resistant *A. baumannii*, particularly XDR and PDR, was associated with a high mortality rate. Septic shock, high SAPS II, high SOFA score, and inappropriate initial antibiotic treatment were independent prognostic factors for 30-day mortality.

## Background

*Acinetobacter baumannii* has become an increasingly significant cause of ventilator-associated pneumonia (VAP) in intensive care units (ICU) that is related to high morbidity and mortality. Recent studies report that *A. baumannii* has emerged as a multidrug-resistant (MDR) organism moving toward extensive drug-resistance (XDR) especially in Asian countries [1,2]. The ICU mortality rate of VAP ranged from 45.6% to 60.9% and has been found to be as high as 84.3% when VAP was caused by XDR *A. baumannii* [3,4]. In our institute, *A. baumannii* was the most common causative pathogen of VAP. We found a rising incidence of VAP caused by XDR *A. baumannii* since 2007 and reported the first case of VAP caused by pandrug-resistant (PDR) *A. baumannii* in our medical ICU in 2010.

Data regarding prognostic factors of mortality among VAP caused by drug-resistant *A. baumannii* in our institute were limited. Therefore, the aims of this study were to identify the prognostic factors of 30-day mortality and to compare survival outcomes of patients with VAP caused by MDR, XDR, and PDR *A. baumannii*.

## Methods

### Study design

A retrospective cohort study was conducted in the 40-bed Medical ICU of Chiang Mai University Hospital. Our hospital is a 1,400-bed, tertiary care university hospital in Thailand. All adult patients diagnosed with VAP caused by *A. baumannii* according to 2005 ATS/IDSA criteria [5] from January, 2005, through December, 2011, were included. Prescription of antibiotics, including drug selection, dosage, and duration of treatment, was guided by our institutional empirical antibiotic guideline for VAP. However, the attending physicians could make decisions for the treatment of VAP by themselves. The study was approved by the Ethics Committee of the Faculty of Medicine, Chiang Mai University.

### Patients

VAP patients with confirmed *A. baumannii* in Medical ICU that were recorded in the infection control surveillance database from 2005 through 2011 were retrospectively reviewed. Criteria for clinical diagnosis of VAP, according to 2005 ATS/IDSA standards [5] were new or progressive pulmonary infiltration which occurred more than 48 h after receiving invasive mechanical ventilation in combination with at least two of three conditions: (1) temperature >38.3°C or <36.0°C, (2) purulent tracheal secretions or a change in characteristics of sputum, or (3) white blood cell count >12,000 or <4,000 cells/mm^3^. VAP was classified by onset of disease as early-onset VAP, which occurred within the first 4 days and late-onset VAP, which developed more than 4 days after receiving mechanical ventilation (MV).

### Microbiological methods

Culture of respiratory specimens, either from bronchoalveolar lavage (BAL) or tracheal aspiration (TA), was used for microbiological diagnosis of VAP. The respiratory pathogens were considered as the causative agents of VAP if they met the diagnostic threshold of either quantitative or semiquantitative culture standard. The quantitative culture was performed by using serial dilution methods. The cut-off points of >10^4^ CFU/ml were used to define positive quantitative culture for BAL [5]. The semiquantitative culture, using the four quadrant method, classified the TA culture results into four categories: 0 = no growth; 1+ = rare growth; 2+ = light or few growth; 3+ = moderate growth; and 4+ = many growth. The report of the culture as moderate to many growth was interpreted as positive by semiquantitative method [6,7].

### Resistant patterns of *A. baumannii*

All patients who met the clinical and microbiological diagnosis of VAP caused by *A. baumannii* were analyzed. The susceptibility of *A. baumannii* isolates to antimicrobial agents was determined using the disk diffusion method. Drug-sensitive (DS) was defined as no resistance to all standard antimicrobial agents. MDR *A. baumannii* was defined as acquired resistance to at least three classes of the following antibiotics: all cephalosporins, aminoglycosides, fluoroquinolones, carbapenems, and beta-lactam/beta-lactamase inhibitors. XDR *A. baumannii* was defined as resistant to all standard antimicrobial agents except colistin or tigecycline. PDR *A. baumannii* was defined as resistance to all categories of antimicrobial agents [8]. Nonmechanical ventilated hospital-acquired pneumonia (HAP) was excluded.

### Data collection

Data was obtained from medical charts and electronic records. Demographic data, including sex, age, and comorbidities, were collected. Sepsis status at VAP onset was classified as sepsis, severe sepsis, and septic shock according to the 2012 Surviving Sepsis Campaign [9]. Clinical pulmonary infection score (CPIS), length of hospital stay (LOS), and mechanical ventilation (MV) days prior to VAP onset were recorded. Disease severity at the onset of VAP was assessed by the Simplified Acute Physiology Score (SAPS II) and the Sequential Organ Failure Assessment (SOFA) score. Other bacterial co-pathogens were collected. Initial empirical antibiotic treatment was considered appropriate or inappropriate depending on whether causative pathogens were sensitive or resistant to prescribed antibiotics. We also considered empirical antimicrobial therapy as inadequate if the other co-pathogens were not sensitive to the medications. The time to start antibiotics was classified as early or late if empirical antibiotic was administered within 24 h or after 24 h of VAP onset, respectively. We also evaluated survival outcome over 30-day after VAP onset among groups of DS, MDR, XRD, and PDR *A. baumannii* VAP patients.

#### Follow-up and outcomes

The outcome was overall 30-day mortality. The patient status at ICU and hospital discharge was also evaluated. All patients were followed up for survival status until 30 days after onset of VAP or until death.

#### Statistical analysis

The data was compared between the survival and non-survival group. Categorical variables were analyzed using Fisher’s exact test. Continuous variables were compared using Student’s *t*-test or Wilcoxon rank sum test as appropriate. Univariable and multivariable Cox’s proportional hazard regression were performed to identify the prognostic factors of mortality. The hazard ratio (HR) and its 95% confidence intervals (CI) were estimated. Variables with a *P* < 0.05 in univariable analysis were included in the final multivariable model using enter selection. The survival analysis was used to compare the survival outcome between DS, MDR, XDR, and PDR *A. baumannii*. All *P* values were two-tailed, and a *P* value <0.05 was considered to be statistically significant. All statistical analysis was performed using STATA version 11.0 (Stata Corp LP, College Station, TX, USA).

## Results

### Characteristics and outcomes of VAP caused by *A. baumannii*

**Figure 1:**
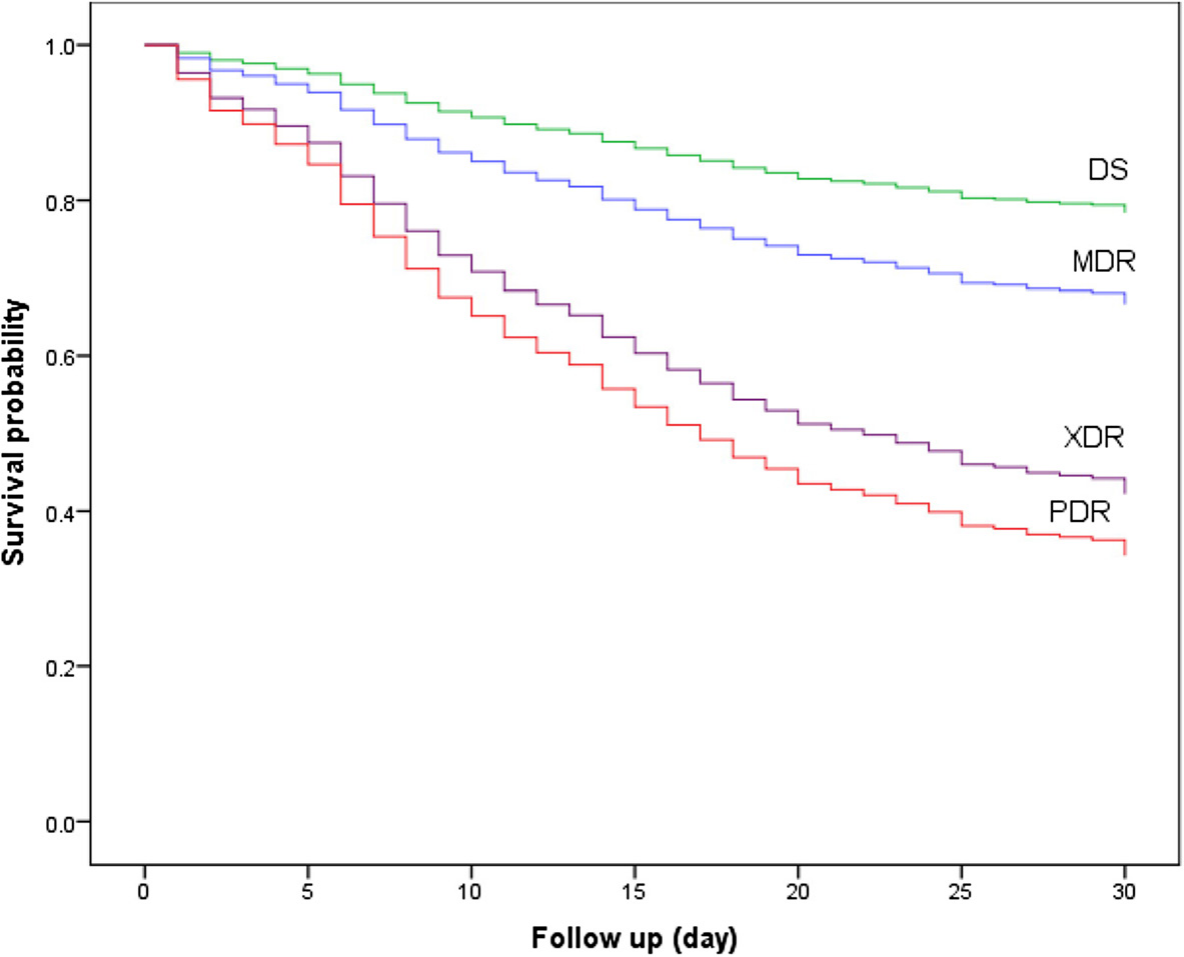
**Survival outcome of patients with VAP caused by DS, MDR, XDR, and PDR** ***A. baumannii.***

A total of 337 patients with microbiologically confirmed *A. baumannii* VAP were reviewed over 7 years. There were 154 (45.7%) males and 183 (54.3%) females with a mean age of 61.6 ± 18.0 years. Late-onset VAP was seen in 88.6% of the patients. The CPIS was 8.2 ± 1.4. The SAPS II and SOFA score were 46.3 ± 14.2 and 6.7 ± 2.7, respectively. The categories of drug-resistant patterns of *A. baumannii* VAP were DS 33 (9.8%), MDR 72 (21.4%), XDR 220 (65.3%), and PDR *A. baumannii* 12 (3.6%). The 30-day mortality rates were 21.2%, 31.9%, 56.8%, and 66.7%, respectively (Tables 1 and 2). The survival outcome among DS, MDR, XDR, and PDR *A. baumannii* is shown in Figure 1. The median duration of mechanical ventilation and hospitalization before VAP onset was 9 and 10 days, respectively. Although 91.1% of the patients received early initial antibiotic treatment, only 56.1% of them were appropriate (Table 2). The overall 30-day, ICU, and in-hospital mortality rates were 48.4%, 32.0% and 53.3%, respectively (Table 3).

**Table 1:** **Demographic and clinical characteristics of patients with VAP caused by** ***A. baumannii***

**Variables**	**All cases**	**Survivors**	**Non-survivors**	***P*** **value**
	**(** ***n*** **= 337)**	**(** ***n*** **= 174)**	**(** ***n*** **= 163)**	
Male gender, *n* (%)	154 (45.7)	87 (50)	67 (41)	0.101
Age, mean ± SD	61.6 ± 18.0	60.0 ± 20.0	63.5 ± 15.5	0.301
Comorbidity, *n* (%)				
Renal diseases	110 (32.6)	51 (29.3)	59 (36.2)	0.178
Cerebrovascular diseases	92 (27.3)	51 (29.3)	41 (25.2)	0.392
Cardiovascular diseases	94 (27.9)	49 (28.2)	45 (27.6)	0.910
COPD	56 (16.6)	30 (17.2)	26 (16.0)	0.750
Diabetes mellitus (DM)	56 (16.6)	27 (15.6)	29 (17.8)	0.591
Immunocompromised hosts^a^	64 (19.0)	23 (13.3)	41 (25.2)	0.006
Malignancy	47 (13.9)	12 (7.0)	35 (21.5)	<0.001
Hematologic diseases	28 (8.3)	10 (5.7)	18 (11.0)	0.059
Hepatic diseases	28 (8.3)	14 (8.0)	14 (8.6)	0.857
VAP onset, *n* (%)				0.851
Early	38 (11.4)	20 (11.7)	18 (11.0)	
Late	296 (88.6)	151 (88.3)	145 (89.0)	
CXR: extents of infiltration, *n* (%)				0.075
Single lobe	300 (89.0)	160 (92.0)	140 (85.9)	
Multi-lobes	37 (11.0)	14 (80)	23 (14.1)	
CPIS, mean ± SD	8.2 ± 1.4	7.97 ± 1.2	8.5 ± 1.5	0.002
Sepsis status, *n* (%)				<0.001
Severe sepsis	178 (52.8)	143 (82.2)	35 (21.5)	
Septic shock	159 (47.2)	31 (17.8)	128 (78.5)	
Severity score				<0.001
SAPS II, mean ± SD	46.3 ± 14.2	39.0 ± 11.2	54.0 ± 12.7	
SOFA, mean ± SD	6.7 ± 2.7	5.4 ± 2.3	8.2 ± 2.3	<0.001
Admission day before VAP onset, median (IQR)	10 (13)	10 (9)	10 (14)	0.499
MV day before VAP onset, median (IQR)	9 (8)	9 (7)	9 (12)	0.485

*COPD* chronic obstructive pulmonary disease, *CPIS* clinical pulmonary infection score, *MV* mechanical ventilator, *SAPS II* Simplified Acute Physiology Score II, *SOFA* Sequential Organ Failure Assessment, *IQR* interquartile range, *CXR* chest X-ray, *VAP* ventilator-associated pneumonia.

^a^Immunocompromised hosts including SLE, HIV/AIDS, and immunosuppressant.

**Table 2:** **Pathogens and antibiotic treatment of patients with VAP caused by** ***A. baumannii***

**Variables**	**All cases**	**Survivors**	**Non-survivors**	***P*** **value**
	**(** ***N*** **= 337)**	**(** ***N*** **= 174)**	**(** ***N*** **= 163)**	
Pathogens				
Single microbial	180 (53.4)	98 (56.3)	82 (50.3)	0.269
Polymicrobial	157 (46.6)	76 (43.7)	81 (49.7)	
*P. aeruginosa*	76 (22.2)	32 (18.5)	44 (27.0)	0.063
*MRSA*	49 (14.5)	18 (10.3)	31 (19.0)	0.024
*K. pneumoniae*	19 (5.6)	9 (5.2)	10 (6.1)	0.702
Others^a^	29 (8.6)	19 (10.9)	10 (6.1)	0.118
Groups of *A. baumannii*				
DS	33 (9.8)	26 (14.9)	7 (4.3)	<0.001
MDR	72 (21.4)	49 (28.2)	23 (14.1)	
XDR	220 (65.3)	95 (54.6)	125 (76.7)	
PDR	12 (3.6)	4 (2.3)	8 (4.9)	
Antibiotic treatment				0.110
Single antibiotic	120 (36.0)	69 (40.4)	51 (31.5)	
Combined antibiotics	213 (64.0)	102 (59.6)	111 (68.5)	
Time to start antibiotic				0.125
Early (within 24 h)	307 (91.1)	163 (93.7)	144 (88.3)	
Late (>24 h)	30 (8.9)	11 (6.3)	19 (11.7)	
Empirical antibiotic treatment				<0.001
Appropriate	212 (62.9)	133 (76.4)	79 (48.5)	
Inappropriate	125 (37.1)	41 (23.6)	84 (51.5)	

*DS* drug-sensitive, *MDR* multidrug-resistant, *XDR* extensively drug-resistant, *PDR* pandrug-resistant *A. baumannii*, *MRSA* methicillin-resistant *Staphylococcus aureus.*

^a^Others including *E. coli*, *S. maltophilia*, *H. influenzae*, methicillin-sensitive *Staphylococcus aureus*, and *Enterococcus* spp.

**Table 3:** **Mortality rate of patients with VAP caused by** ***A. baumannii*** **, classified by drug-resistant patterns**

**Mortality rate**	***A. baumannii***				**All**	***P*** **value**
	**DS (** ***n*** **= 33)**	**MDR (** ***n*** **= 72)**	**XDR (** ***n*** **= 220)**	**PDR (** ***n*** **= 12)**		
30-day, *n* (%)	7 (21.2)	23 (31.9)	125 (56.8)	8 (66.7)	163 (48.4)	<0.001
ICU, *n* (%)	3 (9.1)	10 (13.9)	88 (40.0)	7 (58.3)	108 (32.0)	<0.001
Hospital, *n* (%)	7 (27.3)	32 (44.4)	137 (62.3)	8 (66.7)	178 (53.3)	<0.001

### Co-pathogens and antibiotic treatment

One hundred fifty-seven VAP patients (46.6%) with *A. baumannii* had co-pathogens. The top three co-pathogens were *Pseudomonas aeruginosa* 76 (22.2%), methicillin-resistant *Staphylococcus aureus* (MRSA) 49 (14.5%), and *Klebsiella pneumoniae* 19 (5.6%). Combination antibiotic therapy was administered to 213 patients (64.0%). The most frequently used initial antibiotics were colistin 154 (45.7%), carbapenems 119 (35.3%), fluoroquinolones 74 (22.0%), and cefoperazole/sulbactam 61 (18.1%). The commonly prescribed combination antibiotics, in order of frequency, were colistin plus carbapenems (18.8%), colistin plus vancomycin (13.1%), and carbapenems plus fluroquinolones (11.3%).

### Prognostic factors

The univariable analysis demonstrated that comorbidities with immunocompromised hosts and malignancy, septic shock, SAPS II, SOFA score, and inappropriate antibiotic treatment as well as XDR and PDR *A. baumannii* were significantly associated with 30-day mortality of *A. baumannii* VAP, as shown in Table 4.

**Table 4:** **Prognostic factors of mortality in patients with VAP caused by** ***A. baumannii*** **, univariable Cox’s proportional hazards regression analysis**

**Predictors**	**HR**	**95% CI**	***P*** **value**
Male gender	1.31	0.96–1.79	0.088
Age >60	1.08	0.79–1.48	0.609
Comorbidities			
Renal diseases	1.24	0.89–1.70	0.193
Cerebrovascular diseases	0.85	0.59–1.21	0.365
Cardiovascular diseases	0.95	0.67–1.34	0.790
COPD	0.90	0.60–1.37	0.638
DM	1.13	0.76–1.70	0.541
Immunocompromised hosts	1.68	1.18–2.40	0.004
Malignancy	2.24	1.54–3.27	<0.001
Hematologic diseases	1.50	0.92–2.45	0.102
Hepatic diseases	1.15	0.67–2.00	0.601
Late-onset VAP	1.05	0.64–1.71	0.846
CXR: extents of infiltration			
Multi-lobes	1.41	0.91–2.19	0.128
Septic shock	6.60	4.54–9.60	<0.001
Severity score			
SAPS II >45	5.36	3.71–7.73	<0.001
SOFA >5	8.18	5.20–12.87	<0.001
Admission **≥**7 days before VAP onset	1.18	0.85–1.62	0.309
Pathogens			
Polymicrobial	1.15	0.84–1.56	0.385
*P. aeruginosa*	1.34	0.95–1.90	0.095
MRSA	1.45	0.97–2.14	0.065
*K. pneumoniae*	1.27	0.67–2.41	0.460
Others^a^	0.69	0.36–1.30	0.249
Groups of *A. baumannii*			
DS	1		
MDR	1.75	0.74–4.16	0.119
XDR	3.38	1.58–7.25	0.002
PDR	4.42	1.60–12.20	0.004
Empirical antibiotic treatment			
Combined antibiotics	1.28	0.91–1.78	0.152
Inappropriate	2.30	1.70–3.13	<0.001

*SAPS II* Simplified Acute Physiology Score II, *SOFA* Sequential Organ Failure Assessment, *DS* drug-sensitive, *MDR* multidrug-resistant, *XDR* extensively drug-resistant, *PDR* pandrug-resistant *A. baumannii*, *MRSA* methicillin-resistant *Staphylococcus aureus*, *CXR* chest X-ray*.*

^a^Others including *E. coli*, *S. maltophilia*, *H. influenzae*, methicillin-sensitive *Staphylococcus aureus*, *Enterococcus* spp., *VAP* ventilator-associated pneumonia.

Multivariable analysis indicated that VAP caused by *A. baumannii* patients with presence of septic shock (HR = 2.66, 95% CI 1.71–4.12, *P* < 0.001), SAPS II >45 (HR = 1.58 95% CI 1.01–2.46, *P* = 0.045), SOFA score >5 (HR = 3.33, 95% CI 1.94–5.72, *P* < 0.001), and inappropriate antibiotic treatment (HR = 1.53, 95% CI 1.08–2.20, *P* = 0.016) were significant independent factors associated with 30-day mortality (Table 5).

**Table 5:** **Prognostic factors of mortality in patients with VAP caused by** ***A. baumannii*** **, multivariable Cox’s proportional hazards regression analysis**

**Predictors**	**HR**	**95% CI**	***P*** **value**
Comorbidities			
Immunocompromised hosts	1.29	0.87–1.91	0.915
Malignancy	1.37	0.92–2.06	0.122
Septic shock	2.66	1.71–4.12	<0.001
Severity score at VAP onset			
SAPS II >45	1.58	1.01–2.46	0.045
SOFA >5	3.33	1.94–5.72	<0.001
Groups of *A. baumannii*			
DS	1		
MDR	1.03	0.44–2.45	0.936
XDR	1.64	0.74–3.64	0.220
PDR	1.41	0.47–4.23	0.537
Empirical antibiotic treatment			
Inappropriate	1.53	1.08–2.20	0.016

*SAPS II* Simplified Acute Physiology Score II, *SOFA* Sequential Organ Failure Assessment, *DS* drug-sensitive, *MDR* multidrug-resistant, *XDR* extensively drug-resistant, *PDR* pandrug-resistant *A. baumannii*, *VAP* ventilator-associated pneumonia.

*SAPS II* Simplified Acute Physiology Score II, *SOFA* Sequential Organ Failure Assessment, *DS* drug-sensitive, *MDR* multidrug-resistant, *XDR* extensively drug-resistant, *PDR* pandrug-resistant *A. baumannii*, *VAP* ventilator-associated pneumonia*.*

## Discussion

Drug-resistant *A. baumannii* infections have a high impact on the healthcare setting and are associated with increased morbidity and mortality in critically ill patients who develop VAP. *A. baumannii* was recognized worldwide as one of the most difficult to control nosocomial infections because of its ability to develop antibiotic resistance and survive long periods of time under dry conditions on inanimate objects in the hospital environment [10]. Therefore, the incidence of this organism, particularly drug-resistant *A. baumannii*, was reported to be rising in many countries [4,11-15]. In a recent study of VAP in Asian countries by Chung et al., *Acinetobacter* spp. was the most frequent pathogen (36.5%) followed by *P. aeruginosa* (25.9%) and *K. pneumoniae* (16.8%). Although, the incidence of XDR *Acinetobacter* spp. was high (51.1%) in that study, no PDR *Acinetobacter* spp. was reported [1]. Interestingly, we found that the incidences of XDR (65.3%) and PDR *A. baumannii* 12(3.6%) in our study were higher than other Asian countries. Moreover, this finding was dramatically different from previous studies in Western countries, with no reported VAP caused by PDR *A. baumannii* [16-18].

Moreover, our center is the referral center from all provincial hospitals in Northern Thailand. Not surprisingly, our ICU is crowded with a lot of critically ill patients. In addition, some of our medical ICUs are not cared by intensivists due to an inadequate number of critical care doctors. Those might be the reasons why VAP caused by *A. baumannii*, particularly XDR *A. baumannii*, was still a major problem in our ICU. Due to the high incidence of XDR *A. baumannii* in our center, our local guidelines for empirical antibiotic therapy recommend colistin as the drug of choice for initial treatment of VAP in the Medical ICU. Therefore, the rate of colistin use has increased in our hospital. PDR *A. baumannii* has risen in clinical relevance, perhaps due to the increase in the use of colistin and ability of *A. baumannii* to develop resistance to all antimicrobials, including colistin [19,20].

Although our study showed that polymicrobial infection was not an independent factor of high mortality, outcome in VAP caused by co-infections with *P. aeruginosa* and MRSA trended to be significant in the univariable analysis. Because these organisms are highly virulent, it is unethical to ignore treatment of VAP caused by these organisms. In patients suspected to have multiple infections, we recommend treatment with combined antibiotics at the beginning and adjusting them later after knowing the results of culture and sensitivity tests.

Our findings illustrated that the presence of septic shock, severity of illness with SAPS II >45, and SOFA score >5 at VAP onset and inappropriate empirical antibiotic treatment were independent prognostic factors associated with 30-day mortality.

Previous studies reported that comorbidities, such as malignancy, renal disease, hepatic disease, and immunocompromised hosts; VAP caused by drug-resistant pathogens; and multi-organ failure were prognostic factors of mortality [3,18,21,22]. We also found that malignancy and immunocompromised hosts were prognostic factors in univariable analysis, but they were not independent predictors in multivariable analysis (Table 4).

Septic shock and multiple organ dysfunctions are serious complications of VAP. Presence of shock and high markers of disease severity, such as SAPS II and SOFA scores, were associated with a high mortality [18,23,24]. Our finding, similar to study by Chaari and Lisboa, showed that presence of septic shock at VAP onset was a risk factor of death. In addition, high SAPS II and SOFA scores on the day of VAP onset were also the prognostic factors in our study [3,25].

The level of SAPS II >45 was associated with increased mortality with sensitivity, specificity, and area under the receiver-operation characteristics curve (AUROC) of 76.7%, 77.0%, and 0.77, respectively. Our results regarding a significant level of SAPS II score corresponded to the study by Tejerina [26]. Our study also demonstrated that a SOFA score >5 on the day of VAP onset was a prognostic factor of death with sensitivity, specificity, and AUROC of 86.5%, 73.0%, and 0.80, respectively.

Although late-onset VAP was described to be associated with drug-resistant organisms [5], we found that 86.3% of our patients with early-onset VAP was caused by drug-resistant *A. baumannii*. The likely explanation of this phenomenon was the underlying diseases of the patients that were at risk for healthcare-associated infection. This might explain why the onset of VAP in our study was not associated with a risk factor for mortality.

Mortality was expected to be related to pattern of drug resistance. The 30-day mortality findings of PDR, XDR, and MDR *A. baumannii* in our study were 66.7%, 56.8%, and 31.9%, respectively. In univariable analysis, XDR and PDR *A. baumannii* had a significantly increased risk of death when compared with the DS group (HR 3.38 and HR 4.42, *P* < 0.05). However, similar to previous studies [4,12,27], they failed to demonstrate statistical significance in multivariable analysis. Our study confirmed that appropriate use of antibiotics and severity of VAP was more important than the microbial resistance pattern.

Combination therapy is a controversial issue in management of VAP due to drug-resistant *A. baumannii*. A meta-analysis demonstrated no difference in all-cause mortality between monotherapy and combination antibiotics therapy for MDR, XDR, and PDR *Acinetobacter* infections [28,29]. Appropriate antibiotic therapy is the key factor to improved outcomes for VAP patients. Inappropriate initial antimicrobial therapy was associated with high mortality in many studies [13,30-32]. Our study did demonstrate that inappropriate initial antibiotic therapy was an independent prognostic factor for 30-day mortality by multivariable analysis model.

Our study had some limitations. First, this study was conducted at a single university hospital; the results may not be applied generalized to community hospital. Second, some data, such as onset of septic shock, appropriate administration and modification of antibiotic treatment, and other treatment for sepsis, was incomplete due to the retrospective study design. Further prospective investigations should be conducted. Despite these limitations, this study provides some new data regarding 30-day mortality of drug-resistant *A. baumannii*, especially PDR *A. baumannii* VAP patients.

## Conclusions

VAP caused by XDR and PDR *A. baumannii* had a high mortality rate. Septic shock, high SAPS II, high SOFA score, and inappropriate initial antibiotic treatment were independent prognostic factors for 30-day mortality. For prevention of inappropriate initial antimicrobial therapy in center with a high incidence of drug-resistant *A. baumannii*, empirical antibiotic guidelines based on local surveillance data should be developed. For prevention of development of drug-resistant *A. baumannii*, particularly XDR strain, judicious use of colistin complied with the guideline is recommended.

## References

[1] Chung DR, Song JH, Kim SH, Thamlikitkul V, Huang SG, Wang H (2011). High prevalence of multidrug-resistant nonfermenters in hospital-acquired pneumonia in Asia. Am J Respir Crit Care Med..

[2] Ko KS, Suh JY, Kwon KT, Jung SI, Park KH, Kang CI (2007). High rates of resistance to colistin and polymyxin B in subgroups of *Acinetobacter baumannii* isolates from Korea. J Antimicrob Chemother..

[3] Chaari A, Mnif B, Bahloul M, Mahjoubi F, Chtara K, Turki O (2013). *Acinetobacter baumannii* ventilator-associated pneumonia: epidemiology, clinical characteristics, and prognosis factors. Int J Infect Dis..

[4] Ozgur ES, Horasan ES, Karaca K, Ersoz G, Nayci Atis S, Kaya A (2014). Ventilator-associated pneumonia due to extensive drug-resistant *Acinetobacter baumannii*: risk factors, clinical features, and outcomes. Am J Infect Control..

[5] American Thoracic Society, Infectious Diseases Society of America (2005). Guidelines for the management of adults with hospital-acquired, ventilator-associated, and healthcare-associated pneumonia. Am J Respir Crit Care Med.

[6] Fujitani SC-MM, Tuttle RP, Delgado E, Taira Y, Darby JM (2009). Comparison of semi-quantitative endotracheal aspirates to quantitative non-bronchoscopic bronchoalveolar lavage in diagnosing ventilator-associated pneumonia. Respir Care..

[7] Hashimoto SSN (2013). Evaluation of semi-quantitative scoring of Gram staining or semi-quantitative culture for the diagnosis of ventilator-associated pneumonia: a retrospective comparison with quantitative culture. J Intensive Care..

[8] Magiorakos AP, Srinivasan A, Carey RB, Carmeli Y, Falagas ME, Giske CG (2012). Multidrug-resistant, extensively drug-resistant and pandrug-resistant bacteria: an international expert proposal for interim standard definitions for acquired resistance. Clin Microbiol Infect..

[9] Dellinger RP, Levy MM, Rhodes A, Annane D, Gerlach H, Opal SM (2013). Surviving sepsis campaign: international guidelines for management of severe sepsis and septic shock: 2012. Crit Care Med..

[10] Wendt C, Dietze B, Dietz E, Ruden H (1997). Survival of *Acinetobacter baumannii* on dry surfaces. J Clin Microbiol..

[11] Dent LL, Marshall DR, Pratap S, Hulette RB (2010). Multidrug resistant *Acinetobacter baumannii*: a descriptive study in a city hospital. BMC Infectious Diseases.

[12] Kuo SC, Chang SC, Wang HY, Lai JF, Chen PC, Shiau YR (2012). Emergence of extensively drug-resistant *Acinetobacter baumannii* complex over 10 years: nationwide data from the Taiwan Surveillance of Antimicrobial Resistance (TSAR) program. BMC Infect Dis.

[13] Maragakis LL, Perl TM (2008). *Acinetobacter baumannii*: epidemiology, antimicrobial resistance, and treatment options. Clin Infect Dis..

[14] Richet H, Fournier P (2006). Nosocomial infections caused by *Acinetobacter baumannii*: a major threat worldwide. Infect Control Hosp Epidemiol..

[15] Giamarellou H, Antoniadou A, Kanellakopoulou K (2008). *Acinetobacter baumannii*: a universal threat to public health?. Int J Antimicrob Agents..

[16] Kollef KEGE, Angela RW, Richard MR, Scott TM, Kollef MH (2008). Predictors of 30-day mortality and hospital costs in patients with ventilator-associated pneumonia attributed to potentially antibiotic-resistant gram-negative bacteria. CHEST..

[17] Rello J, Ollendorf DA, Oster G, Vera-Llonch M, Bellm L, Redman R (2002). Epidemiology and outcomes of ventilator-associated pneumonia in a large US database. Chest..

[18] Vincent JL, Rello J, Marshall J, Silva E, Anzueto A, Martin CD (2009). International study of the prevalence and outcomes of infection in intensive care units. JAMA..

[19] Lopez-Rojas R, McConnell MJ, Jimenez-Mejias ME, Dominguez-Herrera J, Fernandez-Cuenca F, Pachon J (2013). Colistin resistance in a clinical *Acinetobacter baumannii* strain appearing after colistin treatment: effect on virulence and bacterial fitness. Antimicrob Agents Chemother..

[20] Bahador A, Raoofian R, Taheri M, Pourakbari B, Hashemizadeh Z, Hashemi FB (2014). Multidrug resistance among *Acinetobacter baumannii* isolates from Iran: changes in antimicrobial susceptibility patterns and genotypic profile. Microb Drug Resist..

[21] Siempos II, Vardakas KZ, Kyriakopoulos CE, Ntaidou TK, Falagas ME (2010). Predictors of mortality in adult patients with ventilator-associated pneumonia: a meta-analysis. Shock..

[22] Marechal H, Layios N, Damas P (2013). The severity of ICU-acquired pneumonia. Curr Infect Dis Rep..

[23] Depuydt PO, Vandijck DM, Bekaert MA, Decruyenaere JM, Blot SI, Vogelaers DP (2008). Determinants and impact of multidrug antibiotic resistance in pathogens causing ventilator-associated-pneumonia. Crit Care..

[24] Bekaert M, Timsit JF, Vansteelandt S, Depuydt P, Vesin A, Garrouste-Orgeas M (2011). Attributable mortality of ventilator-associated pneumonia: a reappraisal using causal analysis. Am J Respir Crit Care Med..

[25] Lisboa T, Diaz E, Sa-Borges M, Socias A, Sole-Violan J, Rodriguez A (2008). The ventilator-associated pneumonia PIRO score: a tool for predicting ICU mortality and health-care resources use in ventilator-associated pneumonia. Chest..

[26] Tejerina E, Frutos-Vivar F, Restrepo MI, Anzueto A, Abroug F, Palizas F (2006). Incidence, risk factors, and outcome of ventilator-associated pneumonia. J Crit Care..

[27] Daniels TL, Deppen S, Arbogast PG, Griffin MR, Schaffner W, Talbot TR (2008). Mortality rates associated with multidrug-resistant *Acinetobacter baumannii* infection in surgical intensive care units. Infect Control Hosp Epidemiol..

[28] Poulikakos P, Tansarli GS, Falagas ME (2014). Combination antibiotic treatment versus monotherapy for multidrug-resistant, extensively drug-resistant, and pandrug-resistant *Acinetobacter* infections: a systematic review. Eur J Clin Microbiol Infect Dis..

[29] Gu WJ, Wang F, Tang L, Bakker J, Liu JC (2014). Colistin for the treatment of ventilator-associated pneumonia caused by multidrug-resistant Gram-negative bacteria: a systematic review and meta-analysis. Int J Antimicrob Agents..

[30] Teixeira PJ, Seligman R, Hertz FT, Cruz DB, Fachel JM (2007). Inadequate treatment of ventilator-associated pneumonia: risk factors and impact on outcomes. J Hosp Infect..

[31] Piskin N, Aydemir H, Oztoprak N, Akduman D, Comert F, Kokturk F (2012). Inadequate treatment of ventilator-associated and hospital-acquired pneumonia: risk factors and impact on outcomes. BMC Infect Dis..

[32] Lee SCHC, Yu TJ, Shieh WB, See LC (2005). Risk factors of mortality for nosocomial pneumonia: importance of initial antimicrobial therapy. Int J Clin Pract..

